# IDENTIFYING COGNITIVE IMPAIRMENT WITH PRIMARY CARE IMPLEMENTATION OF THE BRIEF INTERVIEW FOR MENTAL STATUS

**DOI:** 10.1093/geroni/igz038.2437

**Published:** 2019-11-08

**Authors:** Karen L Gilbert

**Affiliations:** Alzheimer’s Community Care, West Palm Beach, Florida, United States

## Abstract

Overview: An estimated 5.7 million Americans are affected by Alzheimer’s disease (AD; 2018 Alzheimer’s disease facts and figures, 2018, p. 367). Cognitive impairment fails to be identified in the primary care setting as often as 76% of the time (Moyer, 2014, p. 793). Screening can identify patients with emerging impairment who might otherwise appear cognitively intact Grober, Wakefield, Ehrlich, Mabie & Lipton, 2017, p.191). Early identification of cognitive impairment promotes evaluation of treatable causes (Possin et al., 2018, p. 150), and access to early treatment for irreversible disease, facilitating future planning (Swallow, 2017, pp. 57, 63). Methods: This quantitative study’s aim was to identify patients with occult cognitive impairment. After training staff in a Palm Beach County Florida primary care practice, the Brief Interview of Mental Status (BIMS) was administered to patients aged 45 years and older. Results: Seven of 120 screened patients, with no known AD diagnosis, scored as moderately impaired. One of these patients was 64 years of age, the remaining six ranged from age 71 to 93. Fourteen patients scored at the lowest range of “cognitively intact,” eight were under age 65. Conclusion: Cognitive screening of primary care patients with no known diagnosis of AD identified approximately 7% scoring as moderately impaired; an additional 12% scored at the lowest range of “cognitively intact,” suggesting a potentially emerging cognitive impairment warranting follow up evaluation for treatable causes, developing AD, or a related neurocognitive disorder.

